# Altechromone A Ameliorates Inflammatory Bowel Disease by Inhibiting NF-κB and NLRP3 Pathways

**DOI:** 10.3390/md22090410

**Published:** 2024-09-09

**Authors:** Lei Li, Jing Huang, Lixin Feng, Liyan Xu, Houwen Lin, Kechun Liu, Xiaobin Li, Rongchun Wang

**Affiliations:** 1 Biology Institute, Qilu University of Technology (Shandong Academy of Sciences), Jinan 250103, China; ll1061602375@163.com (L.L.); huangjing0126@126.com (J.H.); flixin2022@163.com (L.F.); 15966535461@163.com (L.X.); franklin67@126.com (H.L.); hliukch@sdas.org (K.L.); 2 Engineering Research Center of Zebrafish Models for Human Diseases and Drug Screening of Shandong Province, Jinan 250103, China; 3 Medical Decision and Economic Group, Department of Pharmacy, Ren Ji Hospital, South Campus, School of Medicine, Shanghai Jiaotong University, Shanghai 200030, China

**Keywords:** Altechromone A, zebrafish, anti-inflammatory activity, inflammatory bowel disease, NOD pathway

## Abstract

Altechromone A, also known as 2,5-dimethyl-7-hydroxychromone, is a hydroxyketone containing one hydroxyl and one ketone group. In this study, we isolated Altechromone A from the marine-derived fungus *Penicillium Chrysogenum* (XY-14-0-4). Previous reports show that Altechromone A has various activities including tumor suppression, antibacterial, and antiviral activities. However, there is no study about its anti-inflammatory activity in inflammatory bowel disease (IBD). Here, we assess the anti-inflammatory activity, especially in IBD, and its potential mechanism using the zebrafish model. Our results indicated that Altechromone A has anti-inflammatory activity in a CuSO_4_-, tail-cutting-, and LPS-induced inflammatory model in zebrafish, respectively. In addition, Altechromone A greatly reduced the number of neutrophils, improved intestinal motility and efflux efficiency, alleviated intestinal damage, and reduced reactive oxygen species production in the TNBS-induced IBD zebrafish model. The transcriptomics sequencing and real-time qPCR indicated that Altechromone A inhibited the expression of pro-inflammatory genes including *TNF-α*, *NF-κB*, *IL-1*, *IL-1β*, *IL-6*, and *NLRP3*. Therefore, these data indicate that Altechromone A exhibits therapeutic effects in IBD by inhibiting the inflammatory response.

## 1. Introduction

Inflammatory bowel disease (IBD), mainly including Crohn’s disease (CD) and ulcerative colitis (UC), has become a world-wide healthcare problem. IBD is characterized by an overactive immune response, leading to abnormal inflammation in the digestive system and intestinal tissues [1]. IBD symptoms include rectal bleeding, abdominal pain, diarrhea, and even weight loss [2]. There are various factors, including environmental factors, the invasion of pathogenic microorganisms, and genetic elements, which can lead to IBD [3]. Although there is progress in treatment, IBD is still incurable. Present therapies mainly pay attention to relieving inflammation. An integration of anti-inflammatory medicine, immunomodulators, and biologics is involved in the future treatment of IBD [4,5].

Marine organisms have become a rich source of natural chemicals. Recent years, various chemicals, including alkaloids, polyketides, steroids, terpenes, and peptides, have been found from marine organisms [6]. And these chemical shows various activities, including anti-inflammatory, antitumor, and antioxidant activity [7]. In our lab, Altechromone A, a known compound, was isolated from the deep-sea fungus *Penicillium chrysogenum*. Previous reports show that Altechromone A has various activities such as antiviral, antibacterial, and antitumor activities [8]. However, its anti-inflammatory activity has not been studies. Thus, the anti-inflammatory activity, especially in IBD, was assessed in our experiments.

*Danio rerio* (zebrafish) has been widely used in developmental biology, pharmacological, and toxicological studies, because of its various advantages of having a small size, low cost, transparency, and high genetic similarity to human beings [9,10,11]. As to the immune system, zebrafish has both an innate and acquired immune system, which is similar to that of mammalians [12,13]. Moreover, optical transparency makes it easy to observe the dynamic inflammation process in vivo using transgenic zebrafish, labeling the immune cell with fluorescence protein [14,15,16]. And several inflammatory models have been established by LPS, CuSO_4_, and TNBS [17,18]. Therefore, the zebrafish model was chosen with which to study the anti-inflammatory activity of Altechromone A and its potential mechanism in IBD. 

Here, the zebrafish IBD model was established by 2,4,6-trinitrobenzene sulfonic acid (TNBS) to assess the anti-inflammatory activity of Altechromone A. In our report, the protective effect of Altechromone A was firstly reported.

## 2. Results

### 2.1. The Anti-Inflammatory Activity of Altechromone A in CuSO_4_-, LPS-, and Tail-Cutting-Induced Inflammation

**Figure 1:**
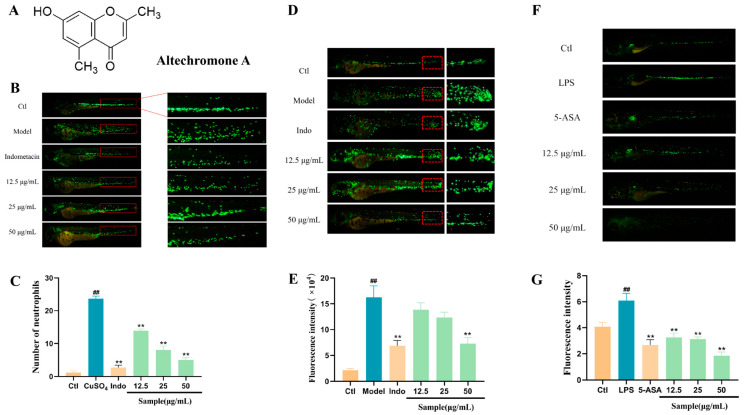
Anti-inflammatory effect of Altechromone A. The results were subjected to analysis using one-way analysis of variance followed by Dunnett’s post hoc *t*-test. ## *p* ≤ 0.01 vs. Ctl, ** *p* ≤ 0.01 vs. model. (**A**) Chemical structure of Altechromone A. (**B**) Effect of Altechromone A on number of neutrophils in CuSO_4_-induced acute inflammation model. (**C**) Images of migration and aggregation of neutrophils, suggested by GFP. The migrated cells were explored in red area. The bar graph counts the number of migrating and aggregated neutrophils. (**D**) Effect of Altechromone A on the fluorescence intensity of neutrophils in tail amputation model. (**E**) Images of migration and aggregation of neutrophils, suggested by GFP. The migrated cells were explored in red area. The bar graph counts the fluorescence intensity (RFU) for migrating and aggregated neutrophils. (**F**) Effect of Altechromone A on the fluorescence intensity of neutrophils in LPS-induced systemic inflammation model. (**G**) Images of migration and aggregation of neutrophils, indicated by GFP. The bar graph counts the fluorescence intensity (RFU) for migrating and aggregated neutrophils.

Acute inflammation can be induced by CuSO_4_ and the immune cells can migrate to the lateral line of the zebrafish. As shown in Figure 1B, compared to the control group, more neutrophils of the model group migrated and were located at the lateral line in the indicated area, while fewer neutrophils in the Indo and Altechromone A groups migrated and were located in the indicated area. 

Mechanical-injury-induced inflammation can be induced by tail amputation. After mechanical damage, the immune cell will migrate to the injury site to repair the damage. As visualized in Figure 1D, more neutrophils migrate to the wound area, while fewer neutrophils were at the cutting area in the Altechromone A groups. 

Lipopolysaccharide (LPS), which is an ingredient of Gram-negative bacteria, can induce systemic inflammation in zebrafish. LPS can result in a notable elevation in the number of neutrophils in the tail (Figure 1F). However, after treatment with Altechromone A, the elevation induced by LPS can be significantly inhibited at concentrations of 12.5–50 μg/mL. These results showed that Altechromone A had remarkable anti-inflammatory activity.

### 2.2. Altechromone A Ameliorated TNBS-Induced Inflammatory Bowel Disease (IBD) by Affecting the Number of Intestinal Leukocytes in Zebrafish

**Figure 2:**
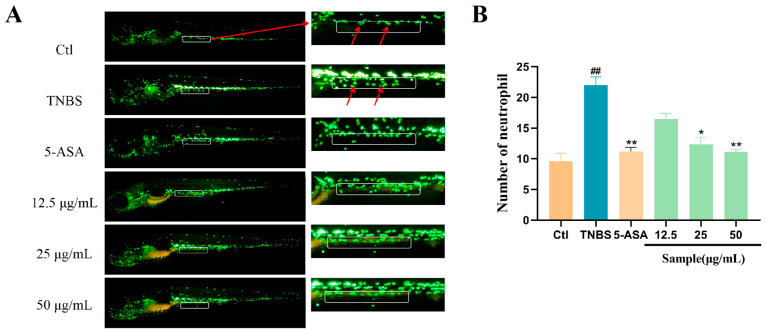
Effect of Altechromone A on the quantity of neutrophils in TNBS-induced IBD model. (**A**) Images of migration and aggregation of leukocytes, suggested by GFP. The migrated cells were explored in white area. (**B**) The quantity of migrating and aggregating leukocytes. The results were subjected to analysis using one-way analysis of variance followed by Dunnett’s post hoc *t*-test. ## *p* ≤ 0.01 vs. Ctl, * *p* ≤ 0.05 vs. model, ** *p* ≤ 0.01 vs. model.

In zebrafish larvae, TNBS can induce enterocolitis characterized by the activation of pro-inflammatory pathways, the disruption of the mucosal barrier, and an increase in leukocyte count in the intestine [19]. As shown in Figure 2A, the TNBS-induced IBD group shows a significant increase in neutrophils in the gastrointestinal area compared to the control group. However, the cotreatment of Altechromone A will decrease the number of neutrophils in the gastrointestinal area compared to the only-TNBS-treatment group. In addition, the anti-inflammatory role of Altechromone A exhibits a dose-dependent behavior (Figure 2B).

### 2.3. Altechromone A Improves Intestinal Function

**Figure 3:**
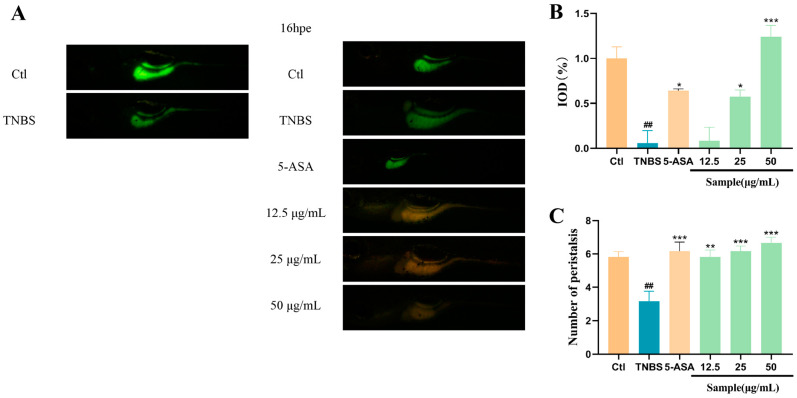
Effect of Altechromone A on intestinal peristalsis and efflux performance in TNBS-induced IBD model. (**A**) Zebrafish was immersed in calcein solution for 10 min, and fluorescent images were photographed at 0 h and 16 h after excretion (16 hpe). (**B**) Quantitation of integrated optical density parameter of the intestinal region. (**C**) Frequency of peristalsis in the intestine after staining. The results were subjected to analysis using one-way analysis of variance followed by Dunnett’s post hoc *t*-test. ## *p* ≤ 0.01 vs. Ctl, * *p* ≤ 0.05 vs. model, ** *p* ≤ 0.01 vs. Model, *** *p* ≤ 0.001 vs. Model.

Calcein can be used for the live staining of zebrafish larvae, where epithelial cells with highly endocytosis and lysosome-rich enterocytes are present in the midgut region. The exocytosis capacity of intestinal cells can be evaluated by the excretion of calcein dye. By comparing with the Ctl group, the excretion ability of zebrafish intestinal segments in the model group was weakened (Figure 3A,B). And the frequency of peristalsis in 1 min was significantly decreased by TNBS, while Altechromone A can promote the peristalsis of the intestine at 25–50 μg/mL Altechromone A (Figure 3C). These results indicated that the excretion ability was improved by Altechromone A. 

### 2.4. The Intestinal Structure Protective Effects of Altechromone A in the TNBS-Induced IBD Zebrafish Model

The results of hematoxylin–eosin (H&E) staining showed that there were more goblet cells distributed in the control groups compared to the TNBS-treated group. However, compared to the TNBS-treated group, more goblet cells were presented in the Altechromone A cotreatment groups. This observation suggests that Altechromone A ameliorated the TNBS-induced damage by protecting the goblet cells, which played a defensive role.

**Figure 4:**
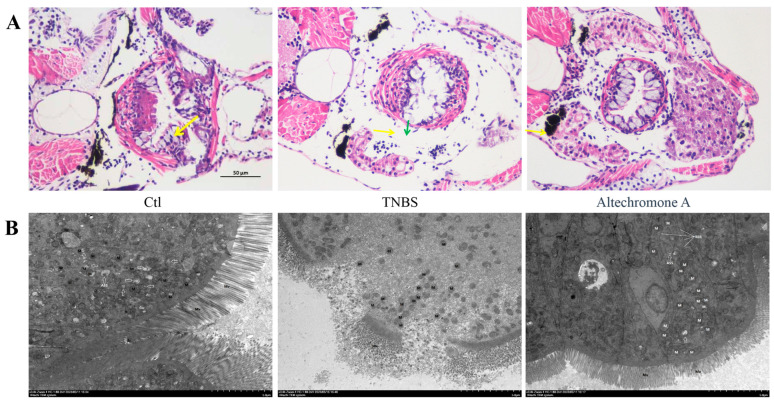
Effect of Altechromone A on intestinal structure in TNBS-induced IBD models. (**A**) Representative H&E-stained cross-sections; the yellow arrow display the goblet cells and the green arrow is the secretion of goblet cells. (**B**) The transmission electron microscope photographs. The results were subjected to analysis using one-way analysis of variance followed by Dunnett’s post hoc *t*-test. Arrows indicate that the rough endoplasmic reticulum (rER) was not significantly expanded, and the ribosomes attached to its surface did not detach.

From the picture of transmission electron microscope, it is obvious that severe edema of the intestinal mucosal epithelial cells, sparse and fragmented microvilli, an abnormal intestinal barrier structure, and noticeable swelling and disruption of mitochondria were observed in TNBS-treated group in comparison to the control group (Figure 4B). Conversely, after Altechromone A treatment, only mild edema of the intestinal mucosal epithelial cells was observed, and the other phenotypes are similar to those of the control group.

### 2.5. Altechromone A Reduced ROS in TNBS-Exposed Larvae

**Figure 5:**
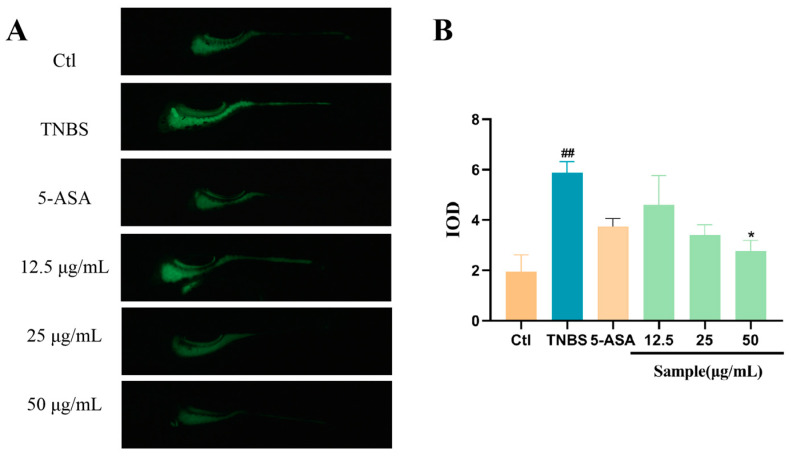
Effect of Altechromone A on content of ROS in TNBS-induced IBD model. (**A**) Images stained with ROS. (**B**) Quantitation of integrated optical density (IOD) parameter of ROS. The results were subjected to analysis using one-way analysis of variance followed by Dunnett’s post hoc *t*-test. ## *p* ≤ 0.01 vs. Ctl, * *p* ≤ 0.05 vs. model.

Since ROS is closely related to the inflammation induced by TNBS, the ROS level was detected after treatment. The ROS level in zebrafish larvae was indicated by the green fluorescence (Figure 5). In the TNBS-exposed group, the content of ROS significantly increased, whereas, the in Altechromone A-treated groups, the content of ROS was notably reduced at concentrations of 12.5, 25, and 50 μg/mL.

### 2.6. Transcriptome Analysis

**Figure 6:**
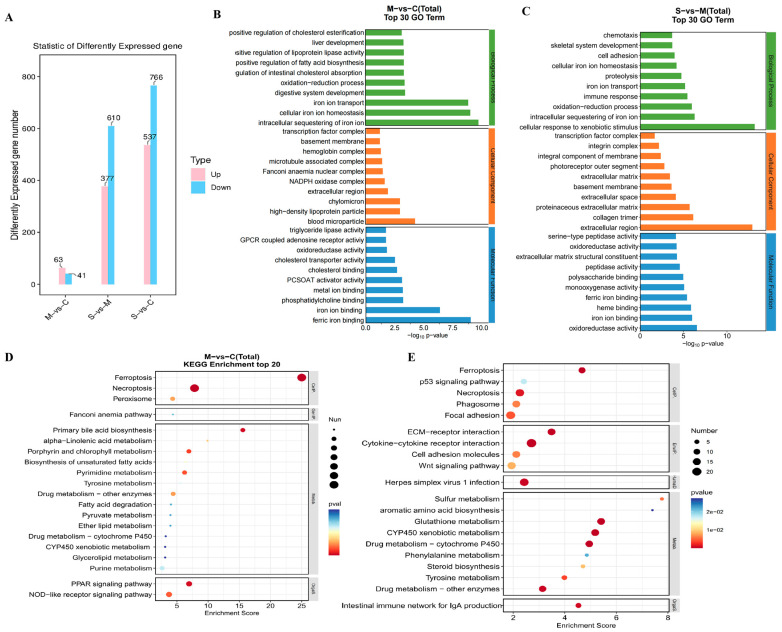
Effect of Altechromone A on transcriptome analysis in TNBS-induced IBD models. (**A**) The histogram of differentially expressed genes. (**B**,**C**) show GO analysis of DEGs of TNBS-induced IBD model and Altechromone A treatment. Green, orange, and blue diagrams showing biological processes, cellular components, and molecular functions. (**D**,**E**) show KEGG enrichment diagram of DEGs of TNBS-induced IBD model and Altechromone A treatment.

To further elucidate the potential mechanism of Altechromone A in treating TNBS-induced IBD in zebrafish, transcriptome sequencing was conducted. First, 50 μg/mL Altechromone A was selected for sequencing since this group has the highest anti-inflammatory activities. The sequence results showed that there were 104 differentially expressed genes (DEGs) (63 upregulated and 41 downregulated genes) between TNBS-treated larval fish and the control group (Figure 6A), and 987 DEGs (377 upregulated and 610 downregulated) at a concentration of 50 μg/mL Altechromone A compared to TNBS-induced larval fish (Figure 6A). 

Subsequently, the subjection of DEGs to a Gene Ontology (GO) functional enrichment analysis was made. In TNBS-induced fish compared to controls, altered biological processes included the response to iron ion transport, cellular iron ion homeostasis, and intracellular sequestering of iron ion (Figure 6B). Conversely, in Altechromone A-administered larval fish, enriched biological processes comprised the cellular response to xenobiotic stimulus, intracellular sequestering of iron ion, and oxidation-reduction processes (Figure 6B). These findings indicate that Altechromone A treatment induces significant changes in gene expression profiles and modulates distinct biological processes compared to both control and TNBS-induced conditions. Notably, Altechromone A appears to influence pathways related to the xenobiotic response and iron homeostasis, which may contribute to its therapeutic effects in TNBS-induced inflammatory bowel disease in zebrafish (Figure 6C).

The outcomes of the top 20 greatly controlled Kyoto Encyclopedia of Genes and Genomes pathways are shown in Figure 6D. Notably, among these pathways, the PPAR signaling pathway and the NOD-like receptor signaling pathway are closely related to inflammation and necroptosis. For Altechromone A, the differentially expressed genes detected belong to the pathways of the p53 signaling pathway, and the intestinal immune network for IgA generation (Figure 6E). These findings suggest that Altechromone A administration influences various pathways related to the immune response, inflammation, and cellular signaling, which may play crucial roles in its therapeutic effects against TNBS-induced inflammatory bowel disease in zebrafish.

### 2.7. Gene Expression

**Figure 7:**
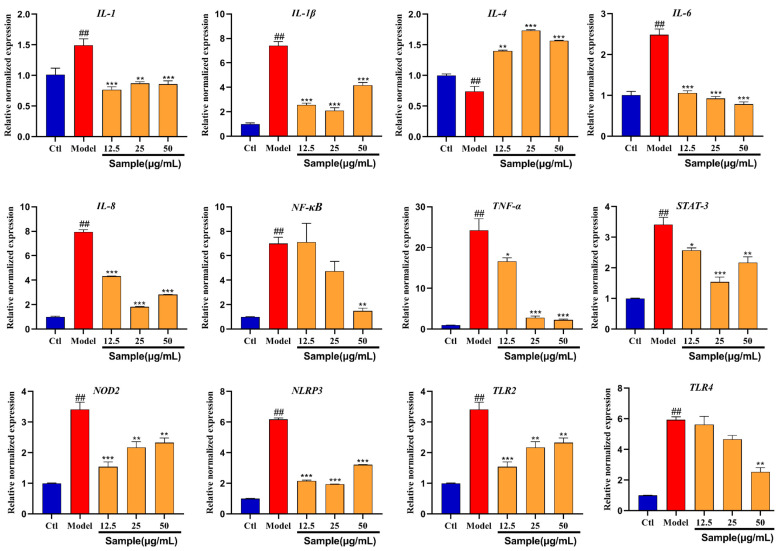
Gene mRNA expression degrees in zebrafish after Altechromone A treatment. The results were subjected to analysis using one-way analysis of variance followed by Dunnett’s post hoc *t*-test. ## *p* ≤ 0.01 vs. Ctl, * *p* ≤ 0.05 vs. model, ** *p* ≤ 0.01 vs. Model, *** *p* ≤ 0.01 vs. model.

In order to study the molecular mechanism of Altechromone A against the inflammatory bowel disease induced by TNBS, the mRNA expression degrees of the genes related to inflammatory bowel disease were evaluated by RT-PCR (Figure 7). The TLR2, TLR4, IL-1β, TNF-α, and NF-κB mRNA expressions of IBD-associated genes were upregulated by TNBS. Nevertheless, the upregulation of genes was inhibited by Altechromone A, especially at the concentration of 50 μg/mL. In addition, the mRNA expression of the IL-4 gene was downregulated by TNBS, while they were inhibited by Altechromone A. 

### 2.8. Altechromone A Attenuates LPS-Induced Inflammation in RAW264.7 Cells

**Figure 8:**
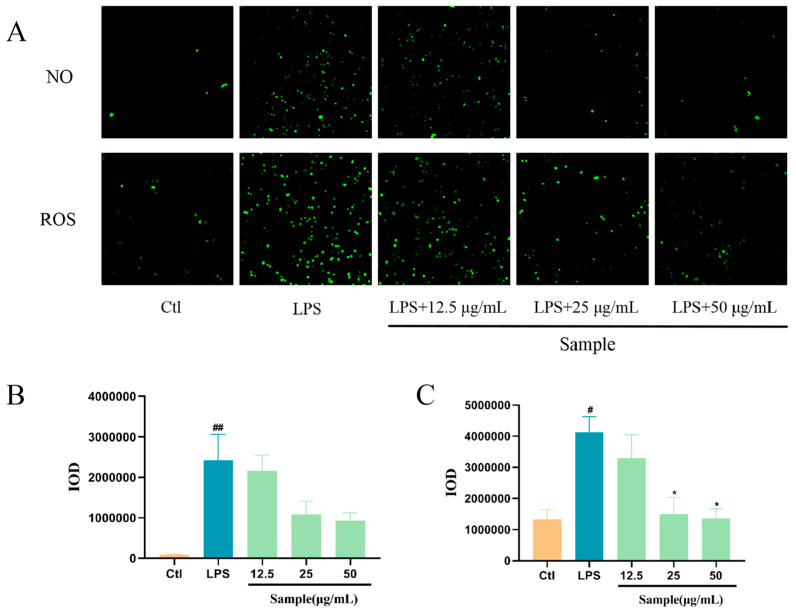
Effect of Altechromone A on NO and ROS levels in LPS-induced RAW264.7 cell model. ## *p* < 0.01 vs. Ctl. (**A**) NO and ROS cell staining images. (**B**) Quantification of the integral optical density (IOD) parameter of NO. (**C**) Quantification of integrated optical density (IOD) parameters for ROS. The results were subjected to analysis using one-way analysis of variance followed by Dunnett’s post hoc *t*-test. # *p* ≤ 0.05 vs. Ctl, ## *p* ≤ 0.01 vs. Ctl, * *p* ≤ 0.05 vs. model.

To further validate the anti-inflammatory effect and the potential mechanism, RAW264.7 cell was used in our studies. Compared to the blank control group, after LPS treatment, RAW 264.7 cells showed significant inflammation and significant increases in both NO and ROS content. After the administration of Altechromone A, the NO and ROS production content was remarkably reduced compared to the model group and showed concentration-dependent behaviors (Figure 8). These results indicated that Altechromone A has anti-inflammatory effects by reducing the NO and ROS levels in LPS-induced RAW264.7 cells.

### 2.9. Protein Expression Level Analysis

**Figure 9:**
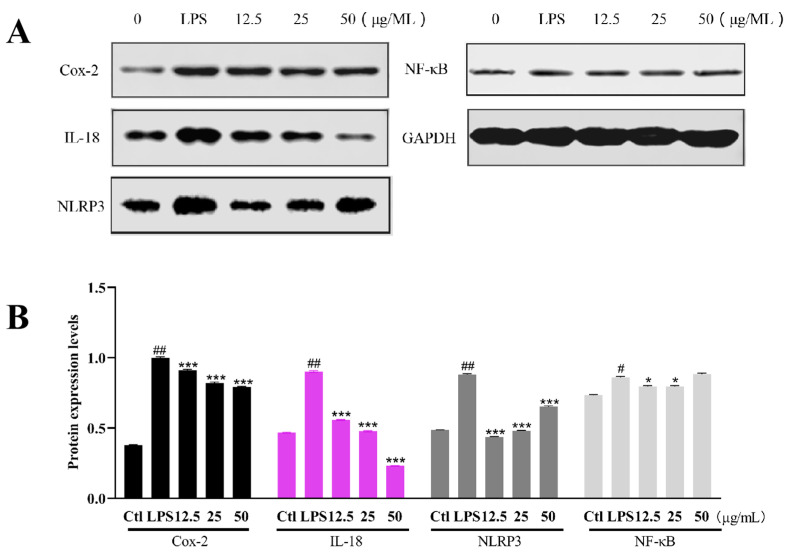
The protein expression level in RAW264.7 cells after 8 h of LPS exposure. (**A**) The protein expression of Cox-2, IL-18, NF-κB, and NLRP3. (**B**) Statistical analysis of protein expression levels. The results were subjected to analysis using one-way analysis of variance followed by Dunnett’s post hoc *t*-test. # *p* ≤ 0.05 vs. Ctl, ## *p* ≤ 0.01 vs. Ctl, * *p* ≤ 0.05 vs. model, *** *p* ≤ 0.001 vs. model.

The results in Figure 9A showed that the expression levels of Cox-2 and IL-18 in the LPS model group were significantly increased compared to the Ctl group, while the expression levels of the proteins in the sample group were downregulated compared to the model group in the concentration-dependent model. The expression levels of NLRP3 were significantly upregulated in the LPS model group, whereas the addition of Altechromone A resulted in a remarkable downregulation. In addition, NF-κB was slightly enhanced in LPS groups compared to the Ctl group, while it is slightly inhibited in the 12.5 and 25 μg/mL Altechromone A groups. These results indicated that Altechromone A could reduce the inflammatory response caused by LPS.

## 3. Discussion

Previous reports show that Altechromone A, derived from Alternaria brassicicola ML-P08, possesses a diverse scope of pharmacological activities such as antiviral, antibacterial, and antitumor characteristics. Gu et al. discovered the potent inhibitory effects of Altechromone A, against Bacillus subtilis, Escherichia coli, Pseudomonas fluorescens, and Candida albicans [20]. To explore the potential therapeutic effects in IBD of Altechromone A, wild-type AB zebrafish lines and the Tg(lyz:EGFP) line in our investigation was adopted. Three zebrafish inflammation models were induced using cupric sulfate, tail amputation and lipopolysaccharide (LPS), and Altechromone A showed excellent anti-inflammatory activities in these three model [21]. In addition, Altechromone A can inhibit anti-inflammatory activities in the IBD zebrafish model induced by TNBS [22]. The excretion ability of zebrafish intestinal segments indicated the function of the intestine. The results of the calcein dye excretion experiment indicated that Altechromone A can relieve the intestine function against TNBS. These results show that Altechromone A possesses protective activities against IBD.

The migration to inflammatory cells is the direct phenomenon of inflammation. The inflammatory response is a protective response to the body or the cells. However, uncontrolled inflammation is associated with many diseases, such as autoimmune diseases, such as rheumatoid arthritis (RA), systemic lupus erythematosus (SLE), IBD, and so on [23]. In addition, chronic inflammation is involved in nearly all other diseases such as cancer, Parkinson’s disease, and cardiovascular and cerebrovascular diseases [24]. There are various factors, including mechanic injury, infection with bacteria and virus, radiation, or exposure of toxic chemicals, which can inducing inflammatory response [25,26]. Our results showed that Altechromone A can inhibit the acute, mechanical-injury-related-, and infectious inflammation induced by CuSO_4_, tail amputation, and lipopolysaccharide (LPS). LPS, a composition of the outer wall of the Gram-negative bacteria cell wall, is a common chemical, used to induce infectious inflammation in mice and zebrafish. These results show that Altechromone A has anti-inflammatory effects.

When the inflammation occurs, the immune cells will migrate to the inflammatory site to repair the injury site. If the inflammation continues, it will cause damage to the body, and auto-immune disease will happen, such as IBD [27]. The intestinal integrity of the mechanical barrier mainly comprises the intestinal epithelial cells and the mucus layer is the first line of defense. The mucus layer is composed of mucin molecules secreted by goblet cells [28]. The results of the electron microscope and the H&E staining showed that Altechromone A can maintain the intestinal structural integrity against the injury caused by TNBS.

Oxidative stress is a main factor in IBD progression. Excessive ROS will break the imbalance of oxidative and antioxidant defenses in the gut, and then result in the progression of the intestinal inflammatory [29]. The ROS level is closely related to the severity of IBD [30]. The overexpression of ROS will lead to DNA damage, lipid metabolic disorders, and even the death of the normal cells. Our study showed that the ROS level can be activated by TNBS which is consistent with previous studies. However, Altechromone A can significantly inhibit the ROS elevation induced by TNBS. The results indicated that the inhibition of excessive ROS production is involved in the protection effect of Altechromone A in IBD. 

To investigate the potential molecular mechanism of Altechromone A against IBD, RNA sequencing and RT-PCR were used to detect the gene expression change after Altechromone A treatment. The TNF-α, IL-1β, TLR2, TLR4, IL-1, IL-6, and IL-8 were upregulated after TNBS induction. On the contrary, treatment with Altechromone A led to the reduced expression of these genes, indicating a mitigating role in TNBS-induced inflammation. The Stat3 and NLRP3 pathways which play key roles in the inflammatory responses were inhibited by Altechromone A, while they are activated by TNBS [31]. TLR (TLR2 and TLR4), a type of transmembrane protein, can recognize the extracellular signals and then activate the NF-κB signal pathway by the transfer of NF-κB protein from the cytoplasm to the nucleus to regulate the downstream inflammatory signal [32]. NF-κB can interact with NLRP3 inflammasome to regulate the inflammatory response. Previous reports showed that pro-inflammatory factor IL-18 play important roles in IBD and is an upstream regulator of NF-κB [33]. Our results indicated the expression of IL18 is significantly inhibited in a dose-dependent manner, indicating the anti-inflammatory activity of Altechromone A. The expression of NF-κB also can be slightly inhibited by Altechromone A. However, the expression of NLRP3 is not dose-dependent; this may be because NLRP3 is more sensitive to Altechromone A than NF-κB. These results indicated that Altechromone A can inhibit the inflammatory response by regulating the NF-κB and NLRP3 pathways.

To sum up, the novel finding was that Altechromone A, isolated from marine fungi *Aspergillus*, can relieve inflammatory bowel disease (IBD). Altechromone A can protect the structure and function of intestines against IBD induced by TNBS, by regulating the NF-κB and NLRP3 pathways. The results suggested that Altechromone A could be promising candidates for further pharmacologic and biosynthetic research.

## 4. Materials and Methods

### 4.1. Zebrafish Maintenance and Embryo Collection

Adult male and female zebrafish were separately kept under standard conditions at 28 °C with 4 h light/10 h dark cycles in an automatic zebrafish housing system. Fish was fed twice daily with live brine shrimp and/or commercial flake fish food. For spawning, healthy and mature male and female zebrafish were chosen and placed into a mating tank at a proportion of 1:2. The next morning, embryos were gathered 1 h after fertilization, stimulated by the light. Then, the embryos were cleaned and incubated in E3 water (5 mM NaCl, 0.17 mM KCl, 0.4 mM CaCl_2_, and 0.16 mM MgSO_4_) before the following experiments.

### 4.2. Fungal Material

Marine fungus *Penicillium Chrysogenum* (XY-14-0-4) was isolated from the seawater sample of the Atlantic Ocean in 2015, and identified through the 18s rDNA sequences (GenBank: KY617053.1). The strain was placed at the Drug Screening Research Laboratory, the Institute of Biology Institute of the Shandong Academy of Sciences.

### 4.3. Preparation of Altechromone A

First, 200 mL of Malt Extract Agar (MEA) medium was used for culturing this fungus at 28 °C on a rotary shaker (150 rpm) for 7 days to obtain the seed culture. Then, seed broth was added to 35,500 mL Erlenmeyer flasks each containing solid rice medium made of rice (65 g) and seawater (100 mL), which was sterilized in advance. The fungal strain was incubated under static condition at room temperature for 85 days. All of the fermented material was cut into small pieces and extracted by EtOAc and MeOH (3 × 8.8 L, respectively). The organic solvent was filtered, combined, and concentrated under reduced pressure to obtain the crude extract (65.4 g). By using a silica gel column chromatography (CC) eluting with PE (petroleum ether) /EtOAc (100:0–0:100, *v*/*v*) and CH_2_Cl_2_/MeOH (100:0-0:100, *v*/*v*), the crude extract was separated into seven fractions, Frs. 1–7. Fr. 3 was subjected to Sephadex LH-20 CC (CH_2_Cl_2_/MeOH 1:1, *v*/*v*) and silica CC (PE/EtOAc 100:0-0:100, *v*/*v*) successively to yield compound Altechromone A (114.2 mg).

Altechromone A: White acicular crystals, ^1^H NMR (400 MHz, DMSO-d_6_) δ_H_: 10.6 s, 6.61 brs, 6.59 s, 5.97 s, 2.64 s, 2.26 s; ^13^C NMR (100 MHz, DMSO-d_6_) δ_C_: 178.2, 163.7, 161.2, 159.2, 141.4, 116.6, 114.3, 110.7, 100.5, 22.4, 19.3. This compound was identified by comparison of the above data with those reported [34].

### 4.4. Effect of Altechromone A on Acute Inflammation Caused by CuSO_4_

CuSO_4_ can induce acute inflammation in zebrafish by damaging neuromast in the zebrafish body [35]. The 3 dpf *Tg(zlyz:EGFP)* zebrafish (10 tails per group in 6-well plate) was used to study the anti-inflammatory activity against CuSO_4_. After 2 h treatment with Indomethacin (20 μM) and Altechromone A (12.5, 25, 50 μg/mL), CuSO_4_ was added and the working concentration is 20 μM for 1 h. The migrated immune cells to certain area of lateral line region were photographed and analyzed. Indomethacin was used as positive control. 

### 4.5. Effect of Altechromone A on Inflammation Caused by Tail Amputation

*Tg(zlyz:EGFP)* transgenic zebrafish larvae at 72 hpf was anesthetized in 0.04% tricaine and tail cutting was performed in 2% agarose-coated petri dishes under a stereo microscope. Then, groups (*n* = 20) were set up as follows: model group, Indomethacin (20 μM), and Altechromone A groups (12.5, 25, 50 μg/mL). The zebrafish without tail cutting served as negative control (Ctl). After 6 h, photography was performed, focusing on the cutting region under an Olympus SZX16 fluorescence microscope. The leukocyte migration and aggregation was used to assess the anti-inflammatory activity against mechanical injury by tail cutting. 

### 4.6. Effect of Altechromone A on Inflammation Caused by LPS

As an endotoxin in the cell walls of Gram-negative bacteria, lipopolysaccharide (LPS) can be widely used for developing zebrafish systemic inflammation. The *Tg(zlyz:EGFP)* was exposed to LPS with/without Indomethacin (20 μM), and Altechromone A groups (12.5, 25, and 50 μg/mL) from 72 hpf to 96 hpf. Then, microscopy was carried out with a fluorescence microscope. 

### 4.7. Effect of Altechromone A on IBD Caused by TNBS

For modeling of inflammatory bowel disease (IBD), zebrafish larvae were exposed with 50 μg/mL TNBS from 72 hpf to 124 hpf [36]. And, then, Altechromone A (12.5, 25, and 50 μg/mL) was added for an additional 24 h. The frequency of peristalsis per minute of the intestine was counted under the microscope. Images of the immune cell migration and aggregation to the intestine region were acquired with a fluorescence microscope and analyzed with ImagePro Plus 5.1.

### 4.8. Effects of Altechromone A on Intestinal Efflux Efficiency in TNBS-Induced Zebrafish IBD Model

TNBS-induced IBD model was established as above. Then, the zebrafish was incubated with Calcein solution for 1.5 h as previous described. Then, the fish were washed with fish water for three times for 5 min. And photography was carried out. Then it was incubated in E3 medium for 16 h in the dark. Image was acquired again. The calculation followed the formulae described previously.

IOD_Ctl_ = [IOD_CTL0_ (mean) − IOD_CTL1_]/IOD_CTL0_ (mean)

IOD_TNBS_ = [IOD_TNBS0_ (mean) − IOD_TNBS1_]/IOD_TNBS0_ (mean)

### 4.9. Histopathological Observations

For assessing the intestinal pathological changes in zebrafish larvae, hematoxylin–eosin (H&E) staining was carried out according to previous method [37]. Briefly, after treatment with the chemicals, the zebrafish larvae were fixed in 4% paraformaldehyde (PFA) at 4 °C next to dehydration by graded ethanol before being embedded in paraffin. Sections were stained with H&E. The images were acquired with Upright optical microscope (Nikon Eclipse E100, Tokyo, Japan). The staining of sections with uranyl acetate and lead citrate was carried out. Transmission electron microscope was adopted to acquire the images (Hitachi HT7800, Tokyo, Japan).

### 4.10. Analysis of ROS

The ROS level was detected using an ROS probe, which can emit fluorescence after reaction with ROS. Brightly, after treatment as in above method, the zebrafish larvae (*n* = 10) was incubated with 20 μg/mL of 2′-7′-Dichlorodihydrofluorescein diacetate (DCF-DA) in the dark at room temperature. Then, tricaine methanesulfonate was adopted to anesthetize the larvae, and microscopy was carried out to capture the images. ImageJ software was employed to calculate the intensity of fluorescence. 

### 4.11. Transcriptomic Analysis

After treatment with Altechromone A, zebrafish larvae were collected and stored at −80 °C. Three replicates of each treatment were subjected to RNAseq and every replicate included 20 embryos. RNA-seq was sequenced by OE Biotech, Inc. (Shanghai, China). The definition of genes with modified *p*-value (padj) < 0.05 and fold change > 1.5 as differential expressed genes (DEGs) was carried out, and the top 20 of them were selected for analysis. OECloud tools were adopted to perform bioinformatic analysis such as Ontology enrichment analysis and Kyoto Encyclopedia of Genes and Genomes analysis.

### 4.12. Real-Time Quantitative PCR (qRT-PCR) of Inflammation-Related Genes

Following the above treatments, the zebrafish were washed in PBS, and FastPure Cell/Tissue Total RNA Isolation Kit V2 was adopted to purify the mRNA. Then, reverse transcription of RNA was performed and quantitative polymerase chain reaction (qPCR) was carried out as previously reported [38]. Relative quantitative analysis of inflammation-related gene expression was carried out using β-actin as housekeeping gene. Primers for all genes are offered by Boshang Bioengineering and listed in Table 1.

### 4.13. Cell Culture, ROS, and NO Detection in RAW264.7 Cells 

Mouse monocyte macrophage cell line (RAW264.7) was preserved in the Drug Screening Laboratory of the Institute of Biology, Shandong Academy of Sciences. After resuscitating the frozen RAW264.7 cells, they were inoculated into DMEM medium containing double antibody and 10% fetal bovine serum, and placed in a constant temperature incubator at 37 °C with 5% CO_2_. The culture medium was changed once every other day, and the cells were passaged when their adherent growth reached 80%~90% of the bottom area of the culture flasks used, and the density of the cells was kept at 8 × 10^4^/mL by counting on a cell counter plate, and 100 µL of each well was transferred to a 96-well cell plate for further incubation for 24 h. Then, the culture medium was removed, and the experimental cells were divided into five groups: the blank group (Ctl group), model group (2 μg/mL LPS), and Altechromone A groups (2 μg/mL LPS+12.5, 25, 50 μg/mL Altechromone A), and four replicates were set up for each concentration, and the cells continued to be cultured for another 8 h. After 8 h, the cells were dosed in accordance with the Biyuntian DAF-FM DA (NO fluorescent probe) kit and the Solebo Active Oxygen Detection kit, and then observed under the cell high-content imaging analysis system to count the fluorescence intensity.

### 4.14. Western Blot

After exposing to LPS for 8 h, RAW264.7 cells were collected, washed 2 times with pre-cooled PBS, and then lysed with RIPA buffer for 30 min. After being centrifuged at 12,000× *g* for 20 min, the supernatant of the cells was collected. Protein concentration was determined by the BCA protein assay kit (Beyotime, Shanghai, China). Then, 30 μg of proteins from each group were subjected to SDS-PAGE gel electrophoresis, membrane transferred, and blocked with 5% skimmed milk in TBST for 1 h. After 1 h of blocking, immunoblotting was carried out with the following primary antibodies—rabbit anti-Cox-2 (1:2000), rabbit anti-NLRP3 (1:2000), rabbit anti-IL-18 (1:2000), rabbit anti-NF-κB (1:2000), and rabbit anti-GAPDH (1:5000)—and incubated overnight at 4 °C. Then, the membranes were incubated with HRP-conjugated secondary antibody (1:10,000) for 2 h at room temperature and rinsed three times with TBST. Protein bands were visualized by the addition of ECL reagent and the blots were densitometrically analyzed using lmageJ1.4.3 software.

### 4.15. Statistical Analysis

The results were subjected to analysis using one-way analysis of variance followed by Dunnett’s post hoc *t*-test, utilizing GraphPad Prism 9.5 software. Data were expressed as mean ± standard error. The definition of statistical significance as a *p*-value of <0.05 was made. 

## Figures and Tables

**Table 1:** Primer sequences.

Gene	Forward (5′-3′)	Reverse (5′-3′)
*β-actin*	AGAGCTATGAGCTGCCTGACG	CCGCAAGATTCCATACCCA
*IL-1*	AGGTGCATCGTGCACATAAG	AAGCTGATGGCCCTAAACAG
*IL-1β*	ATGGCAGAAGTACCTAAGCTC	TGGACACAAATTGCATGGTGAAAGT
*IL-4*	GCCATATCCACGGATGCGACAA	GGTGTTCTTCGTTGCTGTGAGGA
*IL-6*	TCTGCTACACTGGCTACA	ACATCCTGAACTTCGTCTC
*IL-8*	CAAGAACCATTGGGATGAAGGAC	CCTTCAGTAGCCTCTGTCCTTGT
*NF-κB*	CAATGAAATCTCCTGGGTG	CAATGAAATCTCCTGGGTG
*TNF-α*	ATGAGCACAGAAAGCATGATC	TACAGGCTTGTCACTCGAATT
*STAT3*	CAGCAGCTTGACACACGGTA	AAACACCAAAGTGGCATGTGA
*NOD2*	TGCCTCGGGAACAGTAAGAC	GCCGCCCTCTCCATTAAAC
*NLRP3*	AGCCTTCCAGGATCCTCTTC	CTTGGGCAGCAGTTTCTTTC
*TLR2*	GGCTTCTCTGTCTTGTGACC	GGGCTTGAACCAGGAAGACG
*TLR4*	AGACCTGTCCCTGAACCCTAT	CGATGGACTTCTAAACCAGCCA

## Data Availability

The data presented in the current study are available upon request from the corresponding author.
